# A Supercritical Fluid Chromatograph for Physicochemical Studies

**DOI:** 10.6028/jres.094.013

**Published:** 1989

**Authors:** Thomas J. Bruno

**Affiliations:** National Institute of Standards and Technology, Boulder, CO 80303

**Keywords:** diffusion coefficients, supercritical fluid chromatography

## Abstract

A supercritical fluid chromatograph has been designed and constructed to make physicochemical measurements, while retaining the capability to perform chemical analysis. The physicochemical measurements include diffusion coefficients, capacity ratios, partition coefficients, partial molar volumes, virial coefficients, solubilities, and molecular weight distributions of polymers. In this paper, the apparatus will be described in detail, with particular attention given to its unique features and capabilities. The instrument has recently been applied to the measurement of diffusion coefficients of toluene in supercritical carbon dioxide at a temperature of 313 K, and pressures from 133 to 304 bar (13.3–30.4 MPa). The data are discussed and compared with previous measurements on similar systems.

## 1. Introduction

The methods of chromatography have been applied to physicochemical problems such as thermophysical property determination for nearly 30 years [1,2]. This application of chromatography to non-analytical problems stems from an understanding of the physical and chemical processes which are known to occur during chromatographic separations [3]. As an example, since hydrogen bonding can play a role in chromatographic separation, we may apply chromatography to the study of hydrogen bonding thermodynamics [4].

The development of chromatography during the last 80 years can be divided into distinct historical periods [5], each with its innovations, fads and failures. During the current period, we have seen the emergence of supercritical fluid chromatography (SFC, in which the carrier is a fluid held above its critical point). Some properties of a typical supercritical fluid can be seen in table 1. The density of the supercritical fluid is very similar to that of a liquid phase. This property explains the greatly enhanced solvation power of the supercritical fluid with respect to the gas phase. The viscosity of the supercritical phase closely resembles that of the gas phase, thus allowing for easy mass transfer. The thermal conductivity (though not shown in table 1) is also relatively large, as one would expect from density and viscosity considerations. The diffusivity (self-diffusion) of the supercritical phase is intermediate between that of a gas and a liquid. This property gives the supercritical fluid the advantage over liquid-liquid extraction. There are many excellent reviews describing the advantages and applications of SFC, and the reader is referred to these for additional details [6,7].

Most of the applications of SFC found in the literature involve analytical or separation problems. The application to physicochemical studies has been relatively slow due to many experimental difficulties. Nonetheless, SFC has been applied to a number of thermophysical problems by several groups. This has included the measurement of capacity ratios, partition coefficients, binary diffusion coefficients, partial molar volumes, virial coefficients, solubilities, and polymer molecular weight distributions. In this work, the physicochemical supercritical fluid chromatograph has been applied to the measurement of binary diffusion coefficients [8–16]. This diffusion coefficient describes the tendency of the solute to diffuse into the carrier (usually referred to as the solvent).

## 2. Theory

Chromatographic instruments and methodology has been applied to the measurement of binary diffusion coefficients since the early 1960s [17,18]. The theoretical principles of the so-called peak broadening method were formalized by Taylor and Aris [19–23]. Since many authors have treated the theory in full detail, only a brief summary of the main results will be provided here.

A sharply defined spike of solute (δ-function like) introduced into a laminar stream of a carrier flowing in an uncoated tube will be subject to both convective action along the axis of the tube and molecular diffusion in the radial direction. If the tube is assumed to be straight, the mathematical treatment of Taylor and Aris gives a simple expression for the plate height *H*:

$$H=\frac{2D_{12}}{u}+\frac{r^{2}u}{24D_{12}},$$ (1)

where *D*_12_ is the binary diffusion coefficient of the solute into the carrier (solvent), *u* is the linear velocity of the mobile phase solvent, and *r* is the internal radius of the uncoated tube. The plate height *H* is an important and experimentally accessible quantity in chromatography, since it describes the efficiency of the chromatographic system [24]. This quantity is the width of a peak (as designated by its variance, *σ*^2^, in length units as opposed to time units) relative to the distance traversed inside the column or tube (i.e., the length of the tube, *L*):

$$H=\sigma^{2}/L.$$ (2)

In a straight tube, the concentration profile of the solute in the carrier will become Gaussian-like when *H*<0.02 m [25]. The same result may be obtained from the Golay equation (mathematical description of capillary gas chromatography) when one assumes a zero thickness for the stationary phase film (i.e., an uncoated tube) [26]. At the carrier fluid velocities encountered in practice, eq (1) reduces to:

$$H=\frac{r^{2}u}{24D_{12}},$$ (3)

which allows calculation of the binary diffusion coefficient.

## 3. Experimental

**Figure 1:**
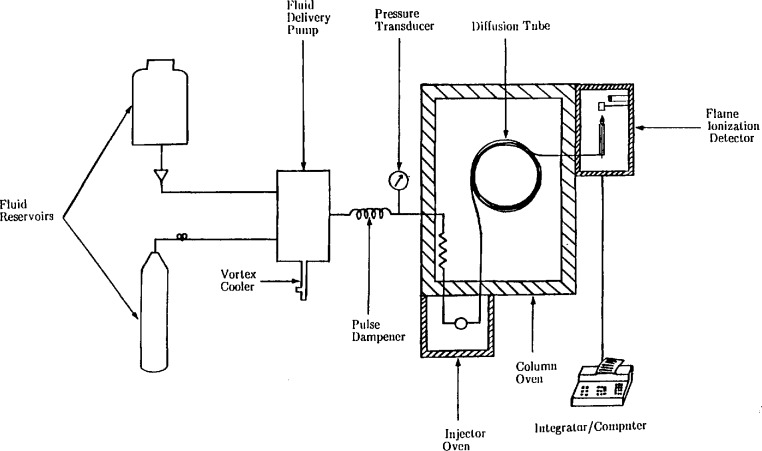
Schematic diagram showing the major components of the supercritical fluid chromatograph used in this work.

The supercritical fluid chromatograph constructed for this work is shown schematically in figure 1. The main components of the instrument are the solvent delivery system, the sample injection system, the column thermostat, and the detection/quantitation system. Safety devices to prevent column temperature and pressure runaway, and inert gas purging of key components provide for explosion-proof operation.

The solvent delivery system is designed to handle fluids which are either gaseous or liquid at room temperature and pressure. The pump is a modified commercial, double piston pressure-controlled device which is electronically compensated for pressure pulsation. This pump is capable of delivering a pressure of 100 MPa, and all associated transfer lines are rated to safely accommodate this limit. The pump head is enshrouded by a welded-seam stainless steel (304) cover (0.16-cm thickness), the inner surface of which is insulated with a 1.27-cm layer of foamed silica. Brazed to the front surface of the cover is a 0.79-cm (inside diameter) compression fitting which accepts the cold stream of a Ranque-Hilsch vortex tube. The vortex tube is used to cool the pump head and carrier stream to facilitate delivery of lower critical point fluids such as carbon dioxide [27,28]. Before the carrier fluid enters the pump head, it is first passed through a heat exchanger which consists of a 600-cm length of stainless steel (304) tubing (0.076-cm inside diameter, 0.16-cm outside diameter). This heat exchanger insures that fluid is delivered to the pump head as a liquid. The vortex tube is operated in an intermediate mode (between maximum cooling and maximum temperature difference, with an applied air pressure of 0.7 MPa), and provides a carrier stream temperature of −20 °C. This minimizes vapor locking and cavitation inside the pump heads.

The pump is followed by a pulse dampener and pressure transducer. The pulse dampener is a coil of flattened stainless steel tubing (0.64-cm outside diameter) which absorbs much of the low level pulsation not handled by the electronic compensation. This component is necessary since no piston pump can operate in true pulse-free fashion. The pressure transducer is a strain gauge device which is calibrated periodically using a high precision Bourdon tube transfer standard. This transfer standard is itself calibrated using a dead weight pressure balance traceable to the NIST primary standard. The uncertainty in the measured pressure has been determined at ±0.40 bar (±0.040 MPa).

**Figure 2:**
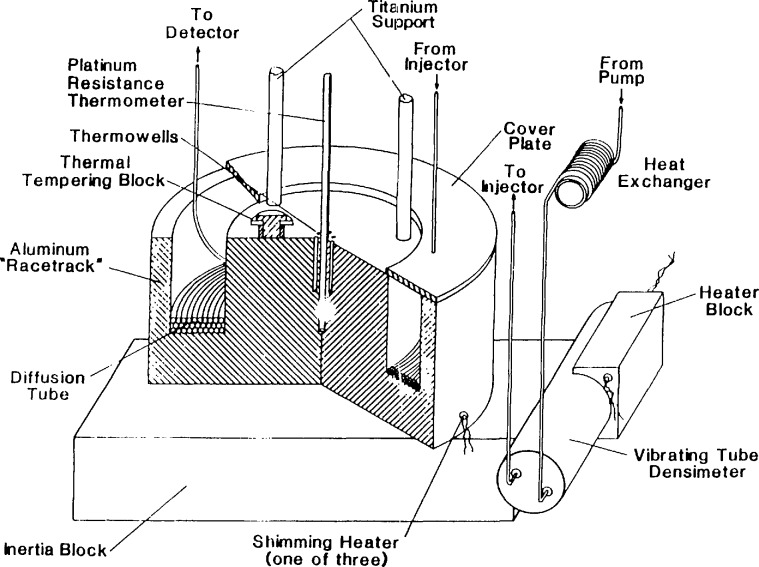
Diagram of tlie thermostatted column area containing the diffusion tube and vibrating tube densimeter.

After leaving the pressure transducer the fluid enters a heat exchanger (fig. 2) inside the column oven, where the flash to supercritical temperature occurs. This heat exchanger is a 300-cm section of stainless steel (304) tubing (0.32-cm outside diameter, 0.07-cm inside diameter). A vibrating tube densimeter is downstream from the heat exchanger, to allow independent density measurements of the carrier if desired. The densimeter places operating constraints upon the entire system (41 MPa maximum pressure, 160 °C maximum temperature), and must be removed for higher temperature or pressure operation.

**Figure 3:**
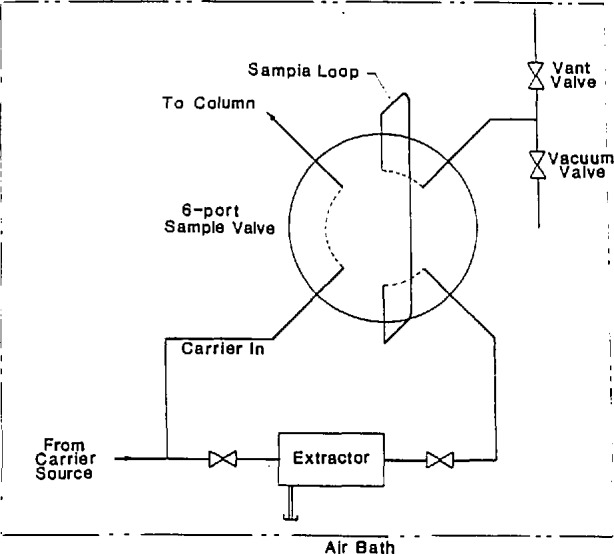
Schematic diagram of the sample injection system.

Following the solvent delivery system is the sample injector. The injector used in this work is the flow-through extractor coupled with a high-pressure chromatographic sampling valve shown in figure 3 [29]. This arrangement is most satisfactory for experiments involving repetitive injections of the same solute. An aliquot of solvent-borne sample is syringe-deposited into the extractor. The extractor is then heated and evacuated to remove the sample solvent. After the solvent is removed (an operation which takes approximately 5 minutes), the extractor is filled with the supercritical carrier (solvent) at the same temperature and pressure as that in the column. The sample is then loaded into the sample loop to allow injection into the carrier stream. The injection is achieved automatically under computer control using an pneumatic actuator (with helium as the working fluid) equipped with pilot valves and a large ballast volume to provide fast switching.

Upon injection, the carrier-borne solute is transported to the column area (fig. 2) inside of a modified commercial forced-air oven capable of maintaining a temperature of 350 °C. The major modifications to the oven include baffles which promote uniform air movement and an inert gas purge line for safety. The column area contains the heart of the physicochemical experiment, which may consist of a coated or uncoated capillary or a packed column. In the case of diffusion coefficient measurements, the column consists of a long uncoated capillary. This capillary is currently a 3040-cm long continuous section of 316 stainless steel tubing (0.159-cm outside diameter, 0.025±0.0013-cm inside diameter) which is connected directly to the injection valve. The length of the diffusion tube was determined by weight, using a calibration equation. This tube is coiled in a 30-cm diameter, held inside of an aluminum racetrack which integrates out local temperature variations. The racetrack also serves as the support for the vibrating tube densimeter referred to earlier. Temperature measurement is provided by a platinum resistance probe located in the center of the racetrack. Six pairs of gradient-sensing thermocouples (type-j, thermally tempered) referenced to the main thermometer provide an indication of temperature nonuniformity. This nonuniformity is then reduced to a negligible level using a set of manually controlled low power shimming heaters. The racetrack is supported inside the oven by titanium rods of 0.64-cm diameter. The low thermal conductivity of the titanium limits heat transfer into or out of the oven. The temperature uniformity of the racetrack has been measured at ±0.015 °C at temperatures at or above 40 °C.

**Figure 4:**
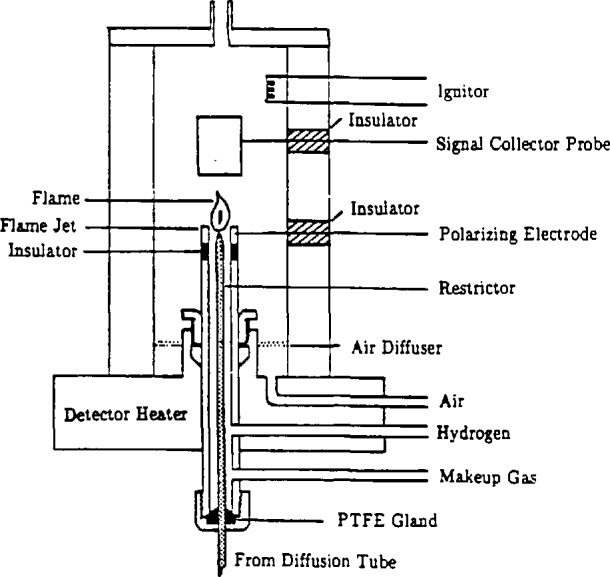
Schematic diagram of the modified flame ionization detector.

After leaving the racetrack, the solute is transported to the detector (fig. 4) by the diffusion tube, as shown in figure 2. The detector is a modified chromatographic flame ionization detector contained in a separate oven directly above the column oven. The major modifications made to the commercial unit were in the sample and jet gas inlet lines. Provisions have also been made for the introduction of a make-up gas where needed. A fused quartz capillary restrictor, attached to the diffusion tube, releases the solute and carrier directly into the flame. The detector is operated to provide an approximate sensitivity of 10^−11^ mol/s. The inside diameter and length of this restrictor capillary is chosen so as to maintain the carrier velocity between 2 and 6 cm/s. The temperature of the detector is maintained at 300 °C to prevent the carrier from cooling (and possibly solidifying) upon decompression. This cooling action has been noted as a cause of baseline “spiking” in unheated detectors. The output from the detector is logged on a commercial electronic integrator, from which the retention times and peak widths may be extracted.

## 4. Results and Discussion

The main sources of error in this experiment stem from the diffusion tube itself, the sample injection process, pressure drop across the tube, and adsorption of the solute on the tube walls. A brief discussion of these errors will be represented here, especially with respect to this apparatus. A number of excellent general reviews are available in the literature [30–34].

The theory of Taylor and Aris, summarized earlier, is strictly based on straight tubes of circular cross-section and uniform inside diameter. For practical reasons the tube is held inside the oven as a helical coil rather than a straight length of tubing. In addition, tubes of perfectly circular cross-section and uniform inside diameter are an idealization not available in the laboratory. We must therefore determine to what extent our experimental apparatus departs from the theoretically assumed conditions. It can be shown [34] that the coiling of the diffusion tube will have no harmful effects if certain criteria are met. The ratio (ω) of the radius of the coil to that of the inside diameter of the diffusion tube should be greater than 100. In the present apparatus, the radius ratio is approximately 1200. Another requirement is that the inequality *De*^2^*Sc* <20 must be satisfied, where *De* is the Dean number and *Sc* is the Schmidt number (see Appendix 1 for definitions). In the design of the present apparatus, *De*^2^*Sc* is always less than 10. Secondary flow effects are minimized by maintaining the carrier velocity below 6 cm/s, thus assuring flow in the laminar regime.

The problem on nonuniformity of the tubing internal radius is somewhat more difficult to control. It is possible to make corrections for this nonuniformity if the nature of the departure is known (for example, a sinusoidal variation superimposed on the radius has been considered [34]). Selection of a good quality seamless tubing will help minimize most of the problems associated with tube nonuniformity. It should be noted that the uncertainty in the tube’s inside diameter cited in the experimental section (~5 percent) is a worst case limit, but this value has been used in the overall error analysis. Since the tube length and radius are both temperature dependent, corrections are applied to account for the effect of thermal expansion on these parameters. The effect of the applied pressure on the internal tube radius is negligible. The effects of connections between the tubes have been minimized by design (only one such connection is used, having the same internal radius as the diffusion tube), and are considered to be negligible.

The errors associated with injection have been addressed in several ways. The sample is introduced at the same pressure and temperature as that of the carrier stream, and at infinite dilution. The volume of the sample loop is very small (≈3.0 *μ*L) as compared with the volume of the diffusion tube. The sampling loop is switched into the carrier stream only for a short time, and then switched back to the fill position. This has been found to decrease problems from solute adsorption. The injection process is done extremely fast using the pilot valve system, resulting in a negligible pressure pulse.

The pressure drop across the diffusion tube has been measured at between 0.3 and 0.6 bar (0.03–0.06 MPa). Since this is on the order of the experimental error of the pressure measurement, no corrections are made, but close proximity of the critical point is avoided. Measurements made within a few degrees of the critical temperature and near the critical pressure will require consideration of this density gradient effect. Solute adsorption on the inside walls of the tube has not been a problem in the present work, as judged from the peak symmetry. Adsorption is often a problem when using highly polar solutes at relatively low carrier density and temperature. To address this issue, diffusion tubes which have larger internal radii are used so as to decrease the surface area-to-volume ratio. This must be done with consideration of the trade-off in radius ratio ω, and the secondary flow effects which may result.

**Figure 5:**
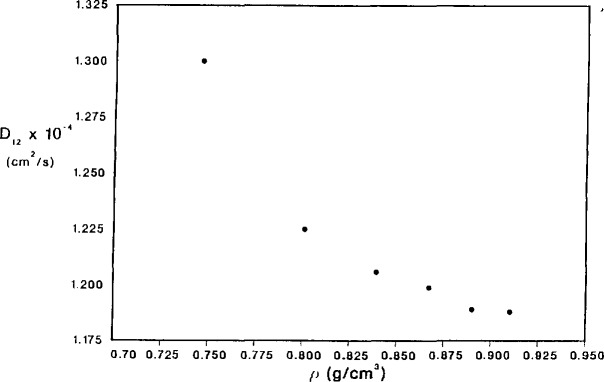
Plot of the binary diffusion coefficient, *D*_12_, of toluene in supercritical carbon dioxide at 313.83 K versus the density of supercritical carbon dioxide.

As an example of the operation of this apparatus, measurements of binary diffusion coefficients of toluene (at infinite dilution) in supercritical carbon dioxide are presented in table 2 [35]. The measurements were made at 313.83±0.02 K, and at pressures from 133 to 304 bar (13.3 to 30.4 MPa). The carrier fluid density corresponding to these temperature-pressure pairs was calculated from the 32-term Benedict-Webb-Rubin (BRW) equation of state for carbon dioxide [36], and ranged from 0.746 to 0.910 g/cm^3^. As discussed earlier, the raw chromatographic data obtained were the peak widths and breakthrough times (the term “retention time” being considered inappropriate due to the absence of a stationary phase). At each density, 15 separate determinations were made, furnishing the experimental uncertainty for the error analysis. The combined uncertainties of all measured quantities provide an overall estimate of between 5 and 6 percent for the data presented here. The diffusion coefficients are all on the order of 10^−4^ cm^2^/s, and decrease with increasing carrier density as can be seen in figure 5. These data fit in quite well with previous data on lower molecular weight aromatics, although most of the previous data were taken at somewhat lower densities [8–11,14]. Comparisons of this data with several predictive approaches are as yet incomplete and will be presented in the future. Current work also includes a study of a homologous series of straight chain hydrocarbons, and several members of the carotene family.

## Figures and Tables

**Table 1:** Comparison of representative fluid properties

	Gas	Liquid	Supercritical fluid
Density, *ρ* g/mL	10^−3^	1	0.7
Diffusivity, *D* cm^2^/s	10^−3^	5×10^−6^	10^−3^
Dynamic viscosity, *η* g/(cm·s)	10^−4^	10^−2^	10^−4^

**Table 2:** Measured binary diffusion coefficients, *D*_12_, of toluene in supercritical carbon dioxide, at 313.83±0.02 K

CO_2_ density (g/mL)	*D*_12_×10^4^ cm^2^/s
0.746	1.30
0.801	1.23
0.893	1.21
0.867	1.20
0.890	1.19
0.910	1.19

## Appendix 1

Some important hydrodynamic parameters:

1.	Radius ratio:	
	ω*=R*_c_*/a*_0_	where *R*_c_ is the overall radius of the coil, and *a*_0_ is the internal radius of the diffusion tube. The effect of diffusion tube coiling may be considered negligible if the radius ratio is greater than 100.
2.	Reynolds number:	
	R_e_=2*a*_0_ *ū*_0_ρ/η	where *ū*_0_ is the average carrier fluid velocity, *ρ* is the carrier density, and *η* is the carrier viscosity. The fluid flow is considered laminar if the Reynolds number is less than 2000.
3.	Schmidt number:	
	*Sc= η/ρ D*_12_	where *D*_12_ is the binary diffusion coefficient.
4.	Dean number:	
	*De*=*Re ω*^−1/2^	The effects of diffusion tube coiling can be considered negligible if *SeDe*^2^<20.
